# Heterologous vaccination with subunit protein vaccine induces a superior neutralizing capacity against BA.4/5‐included SARS‐CoV‐2 variants than homologous vaccination of mRNA vaccine

**DOI:** 10.1002/mco2.238

**Published:** 2023-03-10

**Authors:** Dandan Peng, Tingmei Zhao, Weiqi Hong, Minyang Fu, Cai He, Li Chen, Wenyan Ren, Hong Lei, Jingyun Yang, Aqu Alu, Yanghong Ni, Jian Liu, Jiong Li, Wei Wang, Guobo Shen, Zhiwei Zhao, Li Yang, Jinliang Yang, Zhenling Wang, Yoshimasa Tanaka, Guangwen Lu, Xiangrong Song, Xiawei Wei

**Affiliations:** ^1^ Laboratory of Aging Research and Cancer Drug Target, State Key Laboratory of Biotherapy and Cancer Center, National Clinical Research Center for Geriatrics, West China Hospital Sichuan University Chengdu China; ^2^ Center for Medical Innovation Nagasaki University Nagasaki Japan

**Keywords:** heterologous vaccination, mRNA vaccine, recombinant RBD vaccine, SARS‐CoV‐2

## Abstract

BA.4 and BA.5 (BA.4/5), the subvariants of Omicron, are more transmissible than BA.1 with more robust immune evasion capability because of its unique spike protein mutations. In light of such situation, the vaccination against severe acute respiratory syndrome coronavirus 2 (SARS‐CoV‐2) is in desperate need of the third booster. It has been reported that heterologous boosters might produce more effective immunity against wild‐type SARS‐CoV‐2 and the variants. Additionally, the third heterologous protein subunit booster should be considered potentially. In the present study, we prepared a Delta full‐length spike protein sequence‐based mRNA vaccine as the “priming” shot and developed a recombinant trimeric receptor‐binding domain (RBD) protein vaccine referred to as RBD–HR/trimer as a third heterologous booster. Compared to the homologous mRNA group, the heterologous group (RBD–HR/trimer vaccine primed with two mRNA vaccines) induced higher neutralizing antibody titers against BA.4/5‐included SARS‐CoV‐2 variants. In addition, heterologous vaccination exhibited stronger cellular immune response and long‐lasting memory response than the homologous mRNA vaccine. In conclusion, a third heterologous boosting with RBD–HR/trimer following two‐dose mRNA priming vaccination should be a superior strategy than a third homologous mRNA vaccine. The RBD–HR/trimer vaccine becomes an appropriate candidate for a booster immune injection.

## Graphical abstract

We prepared a Delta full‐length spike protein sequence‐based mRNA vaccine (Figure A, B) and developed a recombinant trimeric receptor‐binding domain (RBD) protein vaccine (Figure C). Later, the mRNA vaccine was injected as the “priming” shot, and the RBD–HR/trimer vaccine was used as a third heterologous booster (Figure D).

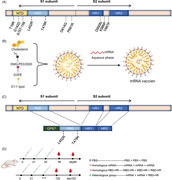

## 1 INTRODUCTION

Since 2019, severe acute respiratory syndrome coronavirus 2 (SARS‐CoV‐2) has caused a pandemic infectious disease called the coronavirus disease 2019 (COVID‐19) and threatened public health considerably. To fight against SARS‐CoV‐2, vaccination has been one of the most effectual tactics so far. Currently, 11 candidate vaccines, based on adenovirus‐based vector, mRNA, protein subunit, and viral vector, have been approved globally against the epidemic. Among them, BNT162B2 (mRNA) from Pfizer‐BioNTech, ChAdOx1 nCoV‐19 (adenovirus‐based vector) from AstraZeneca, mRNA‐1273 (mRNA) from Moderna, and NVX‐CoV2373 (protein subunit) from Novavax are some of the representative vaccines. These COVID‐19 vaccines effectively protected against symptomatic diseases and fatal outcomes arising from the ancestral SARS‐CoV‐2.^1^, ^2^, ^3^, ^4^

Nevertheless, as the continued evolution of SARS‐CoV‐2, the emergence of new variants brings further challenges to the development of vaccines. Notably, the dominant variant, Omicron (B.1.1.529), with a ton of spike protein mutations, was highly transmissible and impaired the population protection conferred by the currently marketed vaccines.^5^ For instance, 2–4 weeks after BNT162B2 vaccination, the neutralizing ability against Omicron variant was reduced 30‐fold compared to the WA1/2020 strain.^6^ Additionally, another study showed a 22‐fold difference in geometric mean titer (GMT) of 50% neutralization between the WA1/2020 strain and Omicron variant (1963 vs. 89).^7^ Recently, a series of lineages of Omicron variants, such as BA.2, BA.3, BA.2.12.1, BA.4, and BA.5 (BA.4/5), have been identified. Among them, the newest members of Omicron, BA.4/5, are dominant in South Africa and the United States and deserve more attention.^8^ Although the spike protein mutations of BA.4/5 are comparable to those of BA.2,^9^ the neutralization escape abilities of BA.4/5 were remarkable increased, which is a more significant obstacle for vaccine development.^10^

In light of this urgent situation, a third booster dose was proposed to be vaccinated for reversing the impaired efficacy of vaccines against Omicron.^11^ Muik et al. found that three doses of BNT162B2 could remarkably increase the neutralizing capacity against Omicron.^12^ Similarly, the homologous mRNA‐1273 booster could also improve the efficacy of the two‐dose mRNA‐1273 vaccination against Omicron variant.^13^ Although the neutralizing capacity could be enhanced by a third booster, the effectiveness against the BA.4/5‐included Omicron variants was still dramatically impaired.^14^ Recent studies implied that a third heterologous booster after two‐dose priming vaccination substantially increased protection. For instance, a third heterologous protein subunit booster after two doses of inactivated whole‐virion vaccines could provide more efficient neutralization than a third homologous inactivated whole‐virion booster.^15^ However, the effects of a third recombinant protein subunit heterologous booster after two doses of mRNA priming vaccines have never, to our knowledge, been evaluated so far.

To investigate whether the heterologous prime‐boost vaccination of recombinant protein vaccine and mRNA vaccine could induce stronger immune response, the NIH mice were injected intramuscularly with two doses of mRNA vaccine, followed by the third dose of receptor‐binding domain (RBD)–HR vaccine. As a control, NIH mice were injected with three doses of PBS, mRNA vaccine, or RBD–HR vaccine, respectively. The vaccinated serum and tissue samples were collected to evaluate vaccination‐induced humoral and cellular immune responses.

## 2 RESULTS

### 2.1 The heterologous vaccine induces higher titers of RBD‐specific binding antibody

**FIGURE 1:**
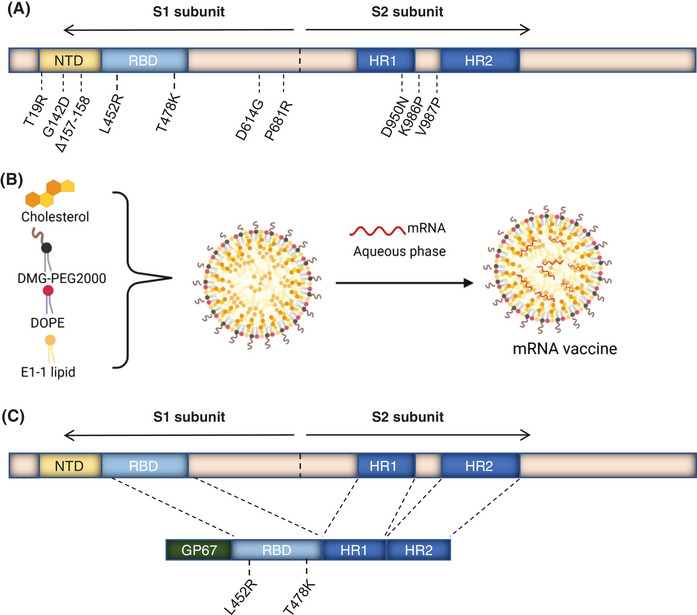
Preparation and characterization of mRNA‐loaded liposome and recombinant receptor‐binding domain (RBD)–HR/trimer protein: (A) the schematic of Delta full‐length spike mRNA construct with mutations; (B) preparation of the mRNA vaccine. Alcohol phases formed by cholesterol, DMG‐PEG2000, DOPE, and E1‐1 lipid were mixed with mRNA aqueous phase at a proper ratio to formulate mRNA vaccine; (C) schematic of the recombinant RBD–HR/trimer protein construct. Our protein comprises an RBD derived from Delta with L452R and T478K mutations, HR1, and HR2 domains. Domains and elements are marked. HR1 and HR2, heptad repeats 1 and 2; NTD, N‐terminal domain; RBD, receptor binding domain. An N‐terminal GP67 signal peptide was designed for secretion. Parts (A) and (B) were created by BioRender.

**FIGURE 2:**
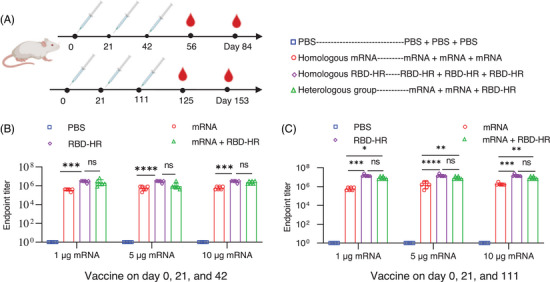
**The schematic diagram and anti‐IgG responses of heterologous and homologous immunization processes**: (A) the schematic diagram of the immunization program. NIH mice were intramuscularly immunized with 1, 5, or 10 µg mRNA vaccine on days 0 and 21. A third heterologous booster, receptor‐binding domain (RBD)–HR/trimer (10 µg), or a homologous booster mRNA vaccine, was vaccinated on day 42, for the long‐term immunization, mRNA vaccine was injected on days 0 and 21, and RBD–HR/trimer booster was given on day 111. mRNA group: three doses of mRNA vaccine; RBD–HR/trimer group: three doses of recombinant RBD–HR/trimer vaccine; mRNA + RBD–HR/trimer group: two doses of mRNA vaccine followed by a heterologous booster with RBD–HR/trimer. The serum samples were collected on day 56 (B) and day 84 (C); (B) the endpoint titers of RBD‐specific IgG in sera collected on day 56 were assayed by enzyme‐linked immunosorbent assay (ELISA); (C) the detection of the titer of anti‐RBD IgG in serum samples collected 125 days after the third dose was performed by ELISA. Part (A) was created by BioRender. The significance of differences among groups was conducted by a One‐way ANOVA analysis followed by Tukey's multiple comparison post hoc test. *p < 0.05; **p < 0.01; ***p < 0.001; ns, not significant.

To investigate whether the third protein subunit heterologous booster could improve the protective efficacy of vaccination over a homologous booster, we designed a Delta full‐length spike protein sequence‐based mRNA vaccine (Figure 1A,B) and developed a recombinant trimeric Delta‐derived RBD protein vaccine (containing L452R and T478K) referred as RBD–HR/trimer^16^ (Figure 1C). The endotoxins of the mRNA vaccine are less than 15 EU/dose or less than 30 EU/mL, and the quality of the RBD/HR vaccine has been approved.^16^ For the broad potential of MF59‐like adjuvant in antibody and T cell responses, MF59‐like adjuvant was selected to serve as an adjuvant to improve the immunogenicity of the RBD–HR/trimer protein.^17^, ^18^ In the present study, the NIH mice were intramuscularly vaccinated with mRNA vaccine on day 0 and 21. After two‐dose vaccination, the mice were immunized with a third‐dose mRNA or RBD‐HR/trimer on day 42 (Figure 2A). To verify the effectiveness of mRNA vaccines, we designed vaccines with three measurements of low (1 µg), medium (5 µg), and high (10 µg). Currently, in most preclinical studies, the COVID‐19 vaccine is administered at intervals of 21 days. To simulate clinical trials, a third dose of the vaccine is given 3 months after the second dose (long‐term immunization). Serum samples were collected on days 56, 84, 115, and 153, and the cytokines were detected within a few days of sampling (Figure 2A). As a control, mice were immunized with three doses of PBS, mRNA, or RBD–HR/trimer (10 µg), respectively. The levels of RBD‐specific lgG titers in serum collected on day 56 (on day 125 for long‐term vaccination) were detected by enzyme‐linked immunosorbent assay (ELISA). As expected, a range dose (1, 5, and 10 µg) of mRNA vaccines elicited high specific anti‐RBD IgG levels (Figure 2B). It is worth noting that the heterologous vaccination with RBD–HR/trimer significantly increased the binding antibody titers (Figure 2B). The trend of binding antibody titers among the long‐term immunization was aligned with the above (Figure 2C). In the subsequent experiment, the serum samples from the mice vaccinated with a 1 or 5 µg dose of mRNA vaccine were utilized.

### 2.2 Heterologous vaccination benefits broad‐spectrum neutralization response against BA.4/5‐included SARS‐CoV‐2 variants

**FIGURE 3:**
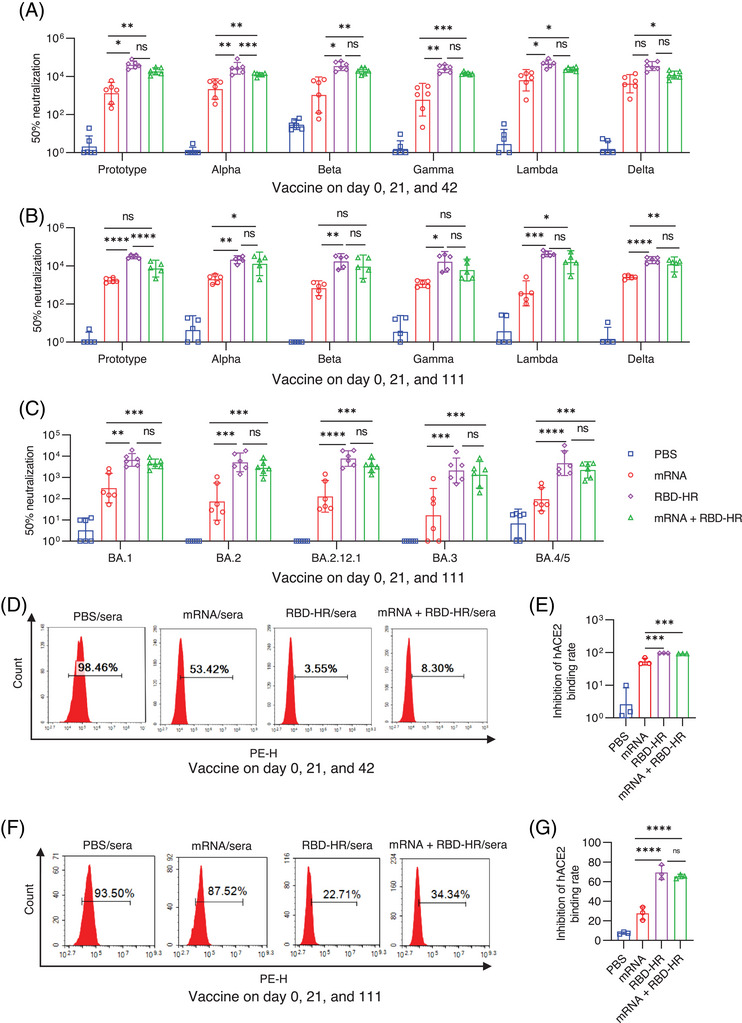
Heterologous group induces stronger broad‐spectrum neutronization response against BA.4/5‐included Omicron variants: (A and B) broad‐spectrum neutronization responses against various pseudoviruses were determined; (C) neutralizing antibody titers against BA.4/5‐included Omicron variants were determined; (D and E) representative images (D) and analysis (E) of flow cytometry represent the blockade of receptor‐binding domain (RBD)‐Fc (Omicron) binding to human ACE2 receptor; (F and G) the flow cytometry images (F) and analysis (G) of the inhibition of RBD‐Fc (Omicron) binding to human ACE2 receptor. NIH mouse were intramuscularly injected with 1 µg mRNA in parts (A)–(C) and 5 µg mRNA in parts (D)–(G) in the homologous mRNA and heterologous groups. Serum samples in parts (A), (C), and (D) were collected on day 84, and serum samples in parts (B) and (F) were collected 153 days after the first dose (long‐term groups). One‐way ANOVA analysis followed by Tukey's multiple comparison post hoc test was conducted in parts (A)–(C), (E), and (G). All error bars represent SEM about the mean. *p < 0.05; **p < 0.01; ***p < 0.001; ****p < 0.0001; ns, not significant.

To evaluate whether the heterologous vaccination could give the better protective efficacy than homologous vaccination, mice were intramuscularly injected with “1 µg” mRNA vaccine in the homologous mRNA group, and sera were collected on day 84 and 153 (long‐term vaccination) since the first immunization, and conducted the pseudovirus neutralization assay (Figure 3A–C). As expected, the homologous mRNA vaccination elicited considerable titers of neutralization antibodies against SARS‐CoV‐2 variants, and the third heterologous vaccination with RBD‐HR/trimer could further provide higher titers of neutralization antibody (Figure 3A). For the homologous mRNA group, the GMTs of 50% neutralization to prototypes, Alpha, Beta, Gamma, Lambda, and Delta, were 2179, 3251, 3122, 1353, 9143, and 5950, respectively. Notably, boosting with the RBD–HR/trimer vaccine significantly increased the neutralizing activities primed by the mRNA vaccine. Compared to the homologous vaccination with mRNA vaccine, the GMTs of 50% neutralization in heterologous vaccination group against prototypes, Alpha, Beta, Gamma, Lambda, and Delta, were observed to increase to 9‐, 3.9‐, 6.5‐, 10.6‐, 2.7‐, and 2.1‐fold, respectively (Figure 3A). Similar results were observed in long‐term heterologous and homologous programs (Figure 3B).

Omicron BA.1 sub‐lineage used to be the dominant variant because of numerous spike protein mutations. Several months later, BA.2 rapidly outcompeted and replaced the BA.1.^19^ Recently, new sub‐lineages of Omicron (BA.2.12.1 and BA.4/5) have become the dominant strains in South Africa and the United States. It has been reported that BA.2.12.1 and BA.4/5 significantly escaped the immune response caused by previous infection or vaccination.^20^, ^21^ To further evaluate the homologous or heterologous vaccination‐induced neutralizing potencies against the Omicron sub‐lineages, sera were collected in the long‐term groups on day 153 for the assay of neutralizing antibody. As expected, the neutralizing capacities induced by homologous mRNA vaccination against various sub‐lineages of Omicron were remarkably impaired. Remarkably, heterologous vaccination with RBD–HR/trimer exhibited stronger neutralization potency against Omicron subvariants, including BA.4/5, and the GMTs of 50% neutralization to BA.1, BA.2, BA.2.12.1, BA.3, and BA.4/5 pseudovirus could reach 4842, 11,284, 3750, 1551, and 2477, respectively. Whereas, in the homologous mRNA group, the GMTs of 50% neutralization determined 786, 796, 809, 734, and 312, respectively (Figure 3C).

For the purpose of verifying the underlying mechanism of viral neutralization, the blockade of binding between cell‐surface ACE2 receptor and RBD‐Fc (Omicron) was detected by the flow cytometry. In‐line with the results of the pseudovirus assay in two immunization programs at different intervals, heterologous vaccination also showed a higher inhibition rate between RBD‐Fc (Omicron) and human ACE2 receptor than homologous mRNA vaccination (Figure 3D–G). Notably, the inhibition rate in the shorter program was better, which underlined the importance of getting booster shots at the proper time (Figure 3D,E). These results demonstrated that the heterologous vaccination with protein subunits could be a better strategy for inducing stronger humoral immunity than the homologous vaccination of mRNA vaccines.

### 2.3 Heterologous vaccine elicits more robust T cell immune response in vivo

**FIGURE 4:**
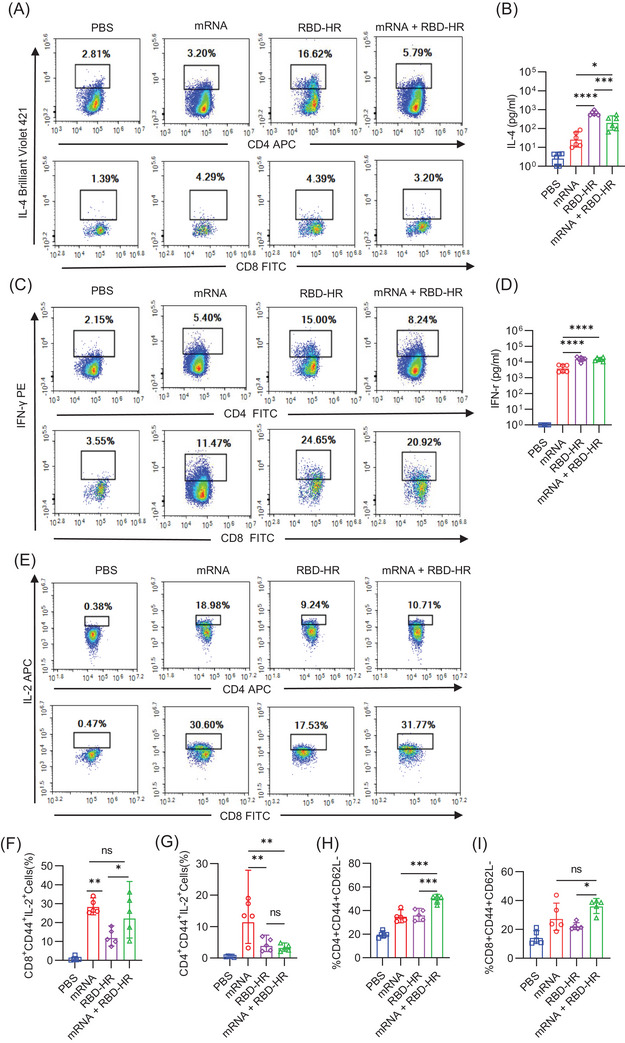
**Heterologous** **vaccine elicits a stronger T cell immune response in vivo**. Lymphocytes were collected from isolated spleens and stimulated with receptor‐binding domain (RBD) (10 µg/mL) proteins for 72 h: (A) representative flow cytometry dot plots of RBD‐specific IL‐4‐producing CD4^+^ (top) and CD8^+^ (bottom) memory T cells (CD44^high^ B220^−^MHC‐II^−^CD4^+^ and CD44^high^ B220^−^MHC‐II^−^CD8^+^); (B) the levels of IL‐4 in cell supernatant were analyzed by enzyme‐linked immunosorbent assay (ELISA); (C) the flow cytometry images of RBD‐specific CD4^+^IFN‐γ^+^ and CD8^+^ IFN‐γ^+^ memory T cells (CD44^high^ B220^−^MHC‐II^−^CD4^+^IFN‐γ^+^ and CD44^high^ B220^−^MHC‐II^−^CD8^+^IFN‐γ^+^); (D) the levels of RBD‐specific IFN‐γ‐producing CD4^+^ (top) and CD8^+^ (bottom) memory T cells; (E ‐ G) The percentage of RBD‐specific IL‐2‐producing memory T cells (CD44^high^ B220^−^MHC‐II^−^CD4^+^IL‐2^+^ and CD44^high^ B220^−^MHC‐II^−^CD8^+^ IL‐2^+^) in the spleen was detected by flow cytometry; (H and I) the percentage of CD4^+^ (G) or CD8^+^ (H) effect memory T cells (CD4^+^CD44^+^CD62L^−^ or CD8^+^CD44^+^CD62L^−^) in lymph node was determined.

T cell responses have an essential effect on preventing SARS‐CoV‐2 and are primary determinants of clinical outcome.^22^ For the homologous mRNA group, mice were intramuscularly injected with 5 µg mRNA. We detected CD4^+^ and CD8^+^ T cell responses against an RBD of SARS‐CoV‐2 in our study. Single‐cell suspension of splenic lymphocytes was prepared to evaluate the further effects of heterologous vaccination with RBD–HR/trimer on the cellular immune response. We detected the secreted cytokines (IL‐4 and IFN‐γ) after being stimulated with the RBD protein at 37°C for 72 h by flow cytometry and ELISA. We found that the levels of secreted IL‐4 (Figure 4A,B) and IFN‐γ (Figure 4C,D) were higher in the heterologous vaccination and homologous protein vaccine group, indicating a strong T cell response could be induced by RBD–HR/trimer vaccine. It was reported that the mRNA vaccine could cause a solid IL‐2‐associated T cell response,^23^ whereas the protein vaccine could not. Notably, the heterologous vaccination and homologous mRNA vaccination induced comparable IL‐2‐associated CD8^+^ T cell response (Figure 4E–G). Memory T cell responses are essential in accelerating and enhancing immune response, especially when reinfected with the same pathogen.^24^, ^25^ In addition, immunological T cell memory response could also provide protective immunity against diverse variants.^26^ We evaluated the subsets of the spleen effector memory (CD44^+^CD62L^−^) T cells on day 49. The number of the CD4^+^ and CD8^+^ effector memory T cells was higher than both mRNA and RBD–HR/trimer homologous vaccination groups (Figure 4H,I). In a word, the heterologous vaccination with RBD–HR/trimer induces stronger cellular immunity than the homologous mRNA vaccine.

### 2.4 Heterologous vaccination induces further long‐lasting memory response

**FIGURE 5:**
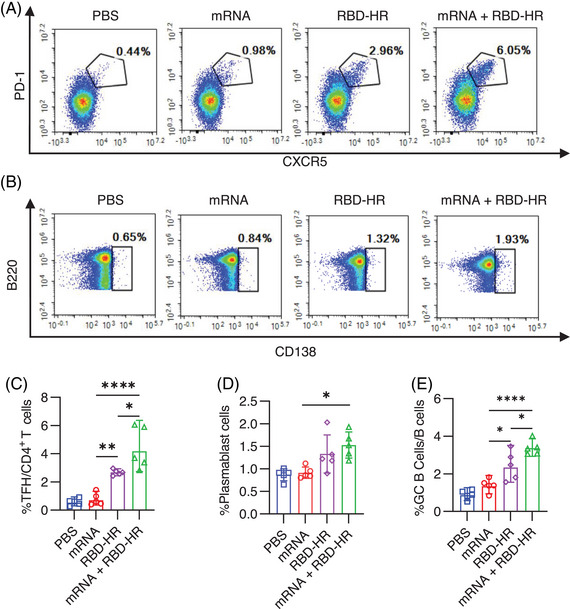
**Heterologous vaccination induces further long‐lasting memory response in NIH mice**: (A) typical pseudocolor flow cytometry plots showing Tfh cells; (B) typical pseudocolor flow cytometry plots showing plasmablast cells; (C–E) frequencies of the Tfh (C), plasmablast (D), and GC B (E) cells in lymph nodes were analyzed. The significance of differences among groups was conducted by a One‐way ANOVA analysis followed by Tukey's multiple comparison post hoc test. *p < 0.05; **p < 0.01; ***p < 0.001; ns, not significant.

Long‐lasting memory response provides rapid and effective protective immunity when reinfected.^27^ For the homologous mRNA group, mice were intramuscularly injected with 5 µg mRNA. To detect whether the heterologous vaccination would induce further memory response with RBD–HR/trimer, we analyzed the T follicular helper (Tfh), plasmablast response, and germinal center (GC) formation, which are essential for the long‐lasting memory response.^28^ Moreover, the GC B and Tfh cells were closely related to neutralizing antibody responses.^18^ The results show that the heterologous vaccination with RBD–HR/trimer could enhance Tfh (Figure 5A,C), plasmablast (Figure 5B,D), and GC B response (Figure 5E). It is worth noting that the frequency of Tfh and GC B cells was the highest in the heterologous vaccination group. Thus, these results suggested that the heterologous vaccination with RBD–HR/trimer could induce a higher magnitude long‐lasting memory immune response than the booster with mRNA vaccine.

## 3 DISCUSSION

BA.2.12.1 and BA.4/5, the emerging sub‐lineages of the B.1.1.529 (Omicron) variant, have displaced as the new dominant strains in South Africa and the United States, and currently marketed vaccines could not provide enough protection against the newly emerged sublineages.^29^ A third boost shot is, therefore, widely requested. The sequential immunization was proposed to elicit vaccine‐induced immune protection, and the heterologous booster could remarkably improve the potency of the vaccination.^30^ In addition, combinations of different vaccines, including ChAdOx1 nCoV‐19 (ChAd)/BNT162b2 (BNT), BNT/ChAd, ChAd/mRNA‐1273, ChAd/BBV152, CoronaVac/BNT, CoronaVac/Convidecia, and Ad26/BNT, have already been validated as well.^31^, ^32^, ^33^, ^34^, ^35^ In addition, the heterologous recombinant protein vaccine booster (ZF2001) after inactivated priming vaccines has already shown dramatically enhancement in anti‐RBD response and neutralizing antibody response.^36^ The underlying mechanism may be associated with the heterologous vaccination‐induced stronger germinal center reactions and a more varied immunoglobulin repertoire.

Nevertheless, as far as we know, the sequential immunization with the combination of mRNA and protein vaccine is still vacant. In the present study, the protein vaccine RBD–HR/trimer was used as the third‐dose heterologous booster after the two‐dose administration of mRNA vaccine. SARS‐CoV‐2 mRNA vaccines could elicit a superior GC response compared to a recombinant protein formulated with an MF59‐like adjuvant (rRBD‐AddaVax) after a single dose.^18^ However, in our study, we found no significance between three doses of homologous RBD–HR/trimer vaccine and homologous mRNA vaccine in GC response. Liao et al. suggested that SARS‐CoV‐2 S‐trimer with MF59‐like adjuvant could also induce GC responses with a low dose.^37^ Both of the homologous groups elicited comparable GC responses, which may benefit from the trimer structure of the RBD–HR protein vaccine.^16^, ^38^, ^39^ Notably, the heterologous RBD–HR/trimer vaccine booster following two doses of mRNA vaccine dramatically enhanced the GC responses.

A recent study suggested that an mRNA‐Omicron booster could not elicit stronger humoral immunity and cellular immunity when compared with an mRNA‐1273 booster for mRNA‐1273‐vaccinated macaques.^40^ In addition, we and others also have shown impaired thermostability and antigenicity of the recombinant Omicron RBD or S1.^41^ Therefore, in our study, we designed the recombinant RBD protein based on Delta rather than Omicron for the effective broad‐variant neutralization of Delta variant. As expected, the heterologous recombinant protein vaccine booster provides enhanced immunity and protection than the homologous mRNA vaccine.

In our research, the third heterologous booster of self‐assembled recombinant RBD–HR vaccine primed with two mRNA vaccines could enhance humoral and cellular immune response, manifested by the higher anti‐RBD IgG, stronger neutralizing antibody, and IFN‐γ‐secreted T cells. Furthermore, the heterologous vaccination could induce stronger effector memory response than both homologous RBD–HR and mRNA vaccinations. In addition, the heterologous vaccination induced a higher plasmablast response than the homologous mRNA group and induced the highest GC B cell and Tfh response than both homologous groups, which indicated that the higher long‐lasting memory response could be elicited by heterologous vaccination with protein vaccine. Therefore, our study provided a solid indication of the heterologous boosting strategy against COVID‐19, and a third heterologous protein subunit booster becomes an appropriate candidate after two doses mRNA vaccine.

## 4 MATERIALS AND METHODS

### 4.1 Materials

The mRNA vaccine we prepared could be translated into the SARS‐CoV‐2 Delta (B.1.617) full‐length spike protein sequence when injected. Recombinant RBD–HR/trimer protein vaccine was developed as previously reported.^16^

### 4.2 Cells

As previously reported,^42^ we generated the 293T cells, which were capable of stably expressing human angiotensin‐converting enzyme 2 receptor (293T/ACE2). The cells were cultured with Dulbecco's modified Eagle's medium (DMEM, Thermo Fisher Scientific, USA) supplied with 10% fetal bovine serum, 0.1 mg/mL Streptomycin and 100 U Penicillin at 37°C with 5% CO_2_.

### 4.3 Animals

NIH female mice aged 6–8 weeks were purchased from Vital River (Beijing, China) and housed in a specific pathogen‐free environment of the State Key Laboratory of Biotherapy.

### 4.4 Vaccine formulation

The alcohol phase (developed by E1‐1 lipid, DOPE, cholesterol, DMG‐PEG2000, and alcohol) and aqueous phases (mRNA sodium citrate solution) were mixed at a proper ratio to prepare the spike‐mRNA vaccine.

We prepared the RBD–HR vaccine as previously.^16^ We mixed MF59‐like adjuvant^43^ and RBD–HR/trimer protein in equal volumes to form the RBD–HR vaccine.^16^

### 4.5 Immunization and samples collection

NIH female mice were randomly divided into four groups, including PBS, homologous mRNA group (mRNA), homologous RBD–HR/trimer group (RBD–HR), and heterologous group (a third RBD–HR/trimer booster following previous priming two doses of mRNA vaccine, mRNA + RBD–HR). Mice were administered according to the homologous or heterologous schedules. The NIH mice were immunized i.m. with a range dose (1, 5, and 10 µg) of mRNA vaccine on days 0 and 21 and immunized i.m. with mRNA or 10 µg RBD–HR vaccine on day 42 after the first vaccination. To test the effects of the RBD–HR vaccine on the immune response for a longer interval after the two doses of mRNA vaccine, we immunized the mice with the third booster on day 111. Blood samples were collected via the eye socket vein on days 56 and 84 and days 125 and 153 and centrifuged at 6000 rpm for 10 min at 4°C. Sera samples were stored at −20°C before use. Seven days after the third‐dose immunization, spleens and lymph nodes were harvested. Spleen tissues and lymph nodes were homogenized and single‐cell suspensions were prepared with a lymphocyte separation medium.

### 4.6 Assay of specific antibodies against SARS‐CoV‐2 RBD

RBD‐specific antibodies in sera were detected by ELISA. Briefly, recombinant RBD proteins (0.1 µg/well) were coated into 96‐well NUNC MaxiSorp plates (Thermo Fisher Scientific, USA) at 4°C for 12 h. Washed the plates with 1× PBST (1× PBS with 0.1% Tween‐20) three times and blocked by 1% bovine serum albumin (BSA) at 37°C for 1 h. The plates were incubated with a 1:2 series of diluted sera samples for 1 h at room temperature and then washed three times by 1× PBST. 1:10,000 diluted horseradish peroxidase (HRP)‐goat‐anti‐mouse IgG antibodies were added to the plates and incubated at room temperature for 1 h with five times wash after that. 3,3′,5,5′‐tetramethyl biphenyl diamine (TMB) was added to the plates and incubated in the dark with 10 min. Stop the reaction by 1 M H_2_SO_4_ (100 µL/well) and determine the absorbance at 450 nm.

### 4.7 Pseudovirus neutralization assay

We purchased variants of SARS‐CoV‐2 pseudoviruses (GFP Luciferase) from Genomeditech Company (China) except BA.3 and BA.4/5 pseudoviruses, which were bought from the Vazyme company (China). NIH mice were intramuscularly injected with mRNA vaccine. After being inactivated at 60°C for 30 min, sera were diluted by a triple‐gradient by complete DME medium. And then, diluted sera were incubated with luciferase‐expressing pseudovirus (prototypes, B.1.1.7, B.1.351, P.1, B.1.617, C.37, BA.1, BA.2, BA.2.12.1, BA.3, and BA.4/5) at 37°C for 1 h. 1.2 × 10^4^ 293T/ACE2 cells were added to each well to express the reporter gene. A period of 48 h later, after removing the infected cell supernatants, 100 µL lysis reagents with luciferase substrate were added to each well. Finally, the plates were detected by a multimode microplate reader (PerkinElmer, USA).

### 4.8 Blockade of RBD‐Fc binding to ACE2 receptor

The binding to the ACE2 receptor was measured by RBD‐Fc (Omicron) protein. Briefly, RBD‐Fc proteins (0.3 µg/mL) were added to sera with serial dilution and incubated at 37°C for 30 min. Then, 293T/ACE2 cells (5 × 10^4^) were added each mixture and incubated at room temperature for 30 min. Cells were washed with BPBS (PBS supplemented with 1% BSA) three times. Then, PE‐labeled antihuman IgG‐Fc antibodies were added to cells and incubated at 4°C in the dark for 30 min. The binding assay was measured by flow cytometry, and the results were analyzed with NovoCyte Flow Cytometer (ACEA Biosciences).

### 4.9 Flow cytometry

For staining Tfh cells, GC B cells, plasmablast cells, and effect memory T cells, the lymphocytes of the spleens and lymph nodes were stained with CD3(PerCP/Cyanine5.5), CD62L(Brilliant Violet 421), CD4(APC), CD8(FITC), CD69(PE‐Cy7), CD44(Brilliant Violet 510), CD95(FITC), B220(PE‐Cy7), GL7(PE), CD4(FITC), CD8(Brilliant Violet 510), CD19(Brilliant Violet 421), CD138(APC), CXCR5(APC), PD1(Brilliant Violet 421), and CD19(PE) (all from BioLegend). Cells were stained in the dark at 4°C. A period of 30 min later, cells were washed by 1× PBS and then detected by flow cytometry.

For intracellular cytokine staining (ICS), the lymphocytes of the spleens were cultured in a 1640 medium for 72 h. Overall, 10% FBS, 100 µg/mL streptomycin, 100 U/mL penicillin, 1 mM pyruvate (all from Gibco, USA), 50 µM β‐mercaptoethanol, and 20 U/mL IL‐2 (all from Sigma‐Aldrich) were added in the 1640 medium. In addition, we added RBD (10 µg/well) to active cells. To block intracellular cytokine secretion, brefeldin A (BFA, BD Biosciences) were incubated for 6 h before staining. Cells were washed in cold 1× PBS and stained with CD4(Brilliant Violet 421), CD8(FITC), CD44(Brilliant Violet 510), B220(PerCP/Cyanine5.5), and MHC‐II(BV711) (all from BioLegend) for 30 min at 4°C. Then cells were fixed and permeabilized to facilitate intracellular staining with anti‐IFN‐γ(PE), anti‐IL‐4(Brilliant Violet 421), and anti‐IL‐2(APC) (all from BioLegend) at room temperature for 2 h. Cells were washed by cold 1× PBS and then detected by flow cytometry.

### 4.10 Statistical analysis

GraphPad Prism version 9.1 was used for Statistical analyses. Comparisons among groups were conducted using One‐way ANOVA followed by Tukey's multiple comparison post hoc test. *p* < 0.05 was considered different. *p < 0.05, **p < 0.01, ***p < 0.001, and ****p < 0.0001 were used to set statistical significance.

## AUTHOR CONTRIBUTIONS

Xiawei Wei, Xiangrong Song, and Guangwen Lu conceived and supervised the research and designed the experiments. Guangwen Lu, Li Yang, and Jiong Li performed gene cloning, expression, and protein purification. Zhengling Wang and Jinliang Yang performed LC–MS/MS analysis to identify glycosylation sites. Wei Wang, Guobo Shen, Zhiwei Zhao, and Zhenling Wang performed the vaccine formulation. Xiangrong Song prepared MF59‐like adjuvant and spike‐mRNA vaccine. Dandan Peng, Mingyang Fu, and Tingmei Zhao performed vaccine formulation and vaccination and performed RDB‐specific binding antibody assay. Dandan Peng, Cai He, Tingmei Zhao, Jingyun Yang, and Hong Lei performed neutralization of pseudovirus experiment, and measurements of blockade of RBD binding to ACE2 receptor. Dandan Peng, Tingmei Zhao, Li Chen, Wenyan Ren, Yanghong Ni, Aqu Alu, and Jian Liu performed flow cytometry to assay T cell immune response and long‐lasting memory responses. Dandan Peng, Mingyang Fu, Weiqi Hong, and Yoshimasa Tanaka analyzed, interpreted the data and assisted with the adjustments of directions and interpretation of the mechanistic aspects of the results Dandan Peng, Mingyang Fu, and Weiqi Hong analyzed, interpreted the data, and wrote the manuscript. All authors have read and approved the final manuscript.

## CONFLICT OF INTEREST STATEMENT

This work was supported by the WestVac Biopharma Co. Ltd. Jiong Li, Wei Wang, Guobo Shen, Zhiwei Zhao, Li Yang, Jinliang Yang, Zhenling Wang, Guangwen Lu, and Xiawei Wei are also working at the WestVac Biopharma Co. Ltd. The remaining authors declare no conflict of interests.

## ETHICS STATEMENT

All animal studies followed and approved by the Institutional Animal Care and Use Committee of Sichuan University (Chengdu, Sichuan, China).

## Data Availability

All the data are available from the corresponding authors upon reasonable request.
